# N6-methyladenosine modification of OIP5-AS1 promotes glycolysis, tumorigenesis, and metastasis of gastric cancer by inhibiting Trim21-mediated hnRNPA1 ubiquitination and degradation

**DOI:** 10.1007/s10120-023-01437-7

**Published:** 2023-10-28

**Authors:** Rongjun Xie, Longfei Liu, Xianzhou Lu, Chengjian He, Hongyi Yao, Guoxin Li

**Affiliations:** 1 Department of General Surgery, Affiliated Nanhua Hospital, Hengyang Medical School, University of South China, Zhuhui District, 336, Dongfeng South Road, Hengyang, 421002 China; 2 Department of General Surgery, Nanfang Hospital, The First School of Clinical Medicine, Southern Medical University, Baiyun District, 1838 Guangzhou Avenue North, Guangzhou, 510515 China; 3 Department of Intensive Care Medicine, Affiliated Nanhua Hospital, Hengyang Medical School, University of South China, Zhuhui District, 336, Dongfeng South Road, Hengyang, 421002 China

**Keywords:** OIP5-AS1, IGF2BP3, HNRNPA1, GC, Growth, Metastasis

## Abstract

**Background:**

Opa-interacting protein 5 antisense transcript 1 (OIP5-AS1) has been demonstrated to play vital roles in development and progression of tumors such as gastric cancer (GC). However, the detailed molecular mechanism of OIP5-AS1 has not been completely elucidated. Our study aimed to investigate the role and the epigenetic regulation mechanism of OIP5-AS1 in GC.

**Methods:**

OIP5-AS1 expression in GC tissues was detected by RT-qPCR. Loss- and gain-of-function experiments were conducted to assess the biological function of OIP5-AS1 in vitro and in vivo. The interaction of OIP5-AS1 with insulin-like growth factor 2 mRNA-binding protein 3 (IGF2BP3) or heterogeneous nuclear nucleoprotein A1 (hnRNPA1) was verified by bioinformatics analysis, RNA pull-down assays, and RNA immunoprecipitation assays.

**Results:**

In this study, we identified that OIP5-AS1 is specifically overexpressed in GC tumor tissues and cell lines and correlated with a poor prognosis. The loss of OIP5-AS1 suppressed the proliferation, migration, invasion, epithelial–mesenchymal transition (EMT), and glycolysis of GC cells, but the ectopic expression of OIP5-AS1 had the opposite impact. Meanwhile, knockdown of OIP5-AS1 inhibited tumor growth in patient-derived xenograft models, as well as repressed tumor metastasis. Mechanistically, IGF2BP3 could bind to OIP5-AS1 by N6-methyladenosine (m6A) modification sites on OIP5-AS1, thereby stabilizing OIP5-AS1. Moreover, OIP5-AS1 prevented Trim21-mediated ubiquitination and degradation of hnRNPA1, stabilizing hnRNPA1 protein and promoting the malignant progression of GC by regulating PKM2 signaling pathway.

**Conclusions:**

In conclusion, this study highlighted that OIP5-AS1 is an oncogenic m6A-modified long non-coding RNA (lncRNA) in GC and that IGF2BP3/OIP5-AS1/hnRNPA1 axis may provide a potential diagnostic or prognostic target for GC.

## Introduction

Gastric cancer (GC) is the most common cancer and the third leading cause of cancer death globally, with 1 million new cases annually [1, 2]. Interestingly, morbidity and mortality rates in Eastern Asia, particularly in China, are dramatically higher [2]. With the advancement of early diagnosis and treatment, incidence and mortality of GC have steadily declined in recent years [3]. Nonetheless, the clinical outcomes of GC patients remain unfavorable due to late diagnosis and tumors [4]. Therefore, identifying viable therapeutic targets for GC treatment is critical.

Long non-coding RNAs (lncRNAs) are a class of newly found non-coding RNA with a length of more than 200 nucleotides [5, 6]. Evidence revealed that aberrant lncRNAs expression exhibits tumor-promotional or tumor-suppressive effects in different cancers, including GC [7, 8]. LncRNAs have been associated with various biological processes, such as cell proliferation, differentiation, apoptosis, inflammation, and tumor metastasis, all contributing to cancer progression [9–15]. For example, lncRNA GMAN reduced GC cell invasive activity and metastasis after injection in GC by binding competitively with GMAN-AS to target ephrin A1 [14]. In another study, the lncRNA UCA1 promotes GC progression by inhibiting SPRY1 and p21 expression by binding with EZH2 [15]. Consequently, studies of key lncRNAs associated with GC are crucial in identifying lncRNAs as potential biomarkers in GC. Recent studies have reported that OIP5 antisense RNA 1 (OIP5-AS1), a kind of lncRNA, plays an oncogenic role in multiple cancers [16]. OIP5-AS1 acts as miRNA sponge and promotes tumor development[17]. For example, OIP5-AS1 promotes cell proliferation and aerobic glycolysis in GC through sponging miR-186[18]. Our previous research showed that downregulation of OIP5-AS1 inhibited GC cell proliferation and colony-formation activity, induced G_0_/G_1_ arrest and apoptosis in vitro, and restrained tumor growth in vivo [19]. Still, the underlying mechanism affecting glycolysis, tumorigenesis, and metastasis of GC in vitro and in vivo remains unknown.

This study aimed to identify OIP5-AS1 as an oncogene. We found that OIP5-AS1 facilitated in vitro proliferation, clone formation, migration, invasion, and glycolysis, as well as in vivo tumor growth and metastasis. OIP5-AS1 stabilized the protein levels of heterogeneous nuclear ribonucleoprotein A1 (hnRNPA1) by regulating the association of ubiquitin E3 ligase tripartite motif-containing protein 21 (TRIM21) with hnRNPA1 to enhance the formation of pyruvate kinase muscle isozyme (PKM) 2. Thus, OIP5-AS1 could serve as a new target for treating GC.

## Materials and methods

### Patient data and tissue sample

GC tissues and the matched corresponding adjacent non-tumor tissues were obtained from 90 patients who underwent surgical treatment at Affiliated Nanhua Hospital, Hengyang Medical School, University of South China from 2018 to 2019 in China. The protocols used in this study were approved by the Ethical Review Committees of Affiliated Nanhua Hospital, Hengyang Medical School, University of South China (No. 2021-KY-156) and written informed consent was obtained from each patient. Clinical parameters, such as age, sex, TNM stage, tumor site, and lymphatic metastasis, were also collected. No patient had received local or systemic treatment before the operation. Fresh samples were frozen in liquid nitrogen immediately after surgical resection and stored at − 80 °C.

### Plasmids, lentivirus, and transfection

The short hairpin RNA (shRNA) against OIP5-AS1 (sh-OIP5-AS1-1, sh-OIP5-AS1-2), OIP5-AS1 overexpression, or their negative control (NC) lentivirus were obtained from Genechem (Shanghai, China) and were infected into GC cells, and screened using 6 μg/mL polybrene. Full-length hnRNPA1 was amplified using PCR and cloned into the pcDNA3.1 vector. SiRNAs targeting IGF2BP3 (si-IGF2BP3), METTL3 (si- METTL3), and hnRNPA1(sihnRNPA1) were synthesized by Genepharma (Shanghai, China). Following the product manuals, GC cells were transfected with the indicated plasmids or siRNAs for 48 h.

### Cell culture

Human normal gastric epithelial cell line (GES-1) and four human GC cell lines (MGC-803, BGC-823, SGC-7901 and AGS) were procured from Jennio Biotech (Guangzhou, China). All cells were maintained in DMEM (Life Technologies, Carlsbad, CA, USA) containing 10% FBS (Sigma Aldrich, St. Louis, MO, USA), 100 U/ml penicillin/streptomycin (Life Technologies) in an atmosphere of 5% CO_2_ at 37 °C.

### Quantitative real-time reverse transcription polymerase chain reaction (qRT-PCR)

The total RNA of tissues and cells was extracted using TRIzol reagent (Invitrogen, Carlsbad, CA, USA). Reverse transcription was performed with the PrimeScript™ RT reagent kit (Applied Biosystems, Foster City, CA, USA). qRT-PCR was implemented on SYBR Select Master Mix and ABI Prism 7000 Sequence Detection System (Applied Biosystems). β-actin was chosen as an endogenous control. Relative expression was quantified according to the 2^−ΔΔCt^ method. Primer sequences are listed in Table 1.

**Table 1:** The sequences of primers and and oligonucleotides were used in this study

Gene	Sequences: 5′-3′
β-actin-F	CACCATTGGCAATGAGCGGTTC
β-actin-R	AGGTCTTTGCGGATGTCCACGT
OIP5-AS1-F	TGCGAAGATGGCGGAGTAAG
OIP5-AS1-R	TAGTTCCTCTCCTCTGGCCG
HNRNPA1-F	ATCTTCCTTTGACCCATTTCC
HNRNPA1-R	AGACCAAGTCCCATCTCAAGC
U6-F	CTCGCTTCGGCAGCACA
U6-R	AACGCTTCACGAATTTGCGT
18S-F	CTGTGATGCCCTTAGATGTCC
18S-R	CCTCGTTCATGGGGAATAATT
sh-OIP5-AS1-1	CCGG-GCTGTGATGCTGGGAACTTAG-CTCGAGCTAAGTTCCCAGCATCACAGC-TTTTTT
sh-OIP5-AS1-2	CCGG-GGAAATGGTGAGCACTCTTAT-CTCGAG-ATAAGAGTGCTCACCATTTCC-TTTTTT
shNC	CCGG-TTCTCCGAACGTGTCACGT-CTCGAG-TTCTCCGAACGTGTCACGT-TTTTTT
siIGF2BP3-sense	GAAACUUCAGAUACGAAAUAU
siIGF2BP3-antisense	AUAUUUCGUAUCUGAAGUUUC
siNC-sense	UUCUCCGAACGUGUCACGU
siNC-antisense	ACGUGACACGUUCGGAGAA
siMETTL3-sense	GAAACUUCAGAUACGAAAUAU
siMETTL3-antisense	AUAUUUCGUAUCUGAAGUUUC
sihnRNPA1-sense	GAAACUUCAGAUACGAAAUAU
sihnRNPA1-antisense	GCCGUGGUGGUGGUGGAUA

### Western blot

Radioimmunoprecipitation assay (RIPA) lysis buffer (Beyotime Biotechnology, Shanghai, China) was used to extract total protein. The protein concentration was measured by a BCA protein assay kit (Thermo Fischer Scientific, Waltham, MA, USA). Equivalent proteins were electrophoresed via 10% sodium dodecyl sulfate–polyacrylamide gel electrophoresis (SDS-PAGE) gel and transferred onto a polyvinylidene fluoride (PVDF) membrane. After membranes were blocked in blocking buffer, it was followed by incubation with primary antibody at 4 °C overnight targeting hnRNPA1 (1:1000; Cell Signaling Technology, Beverly, MA, USA), Trim21 (1:1000; Cell Signaling Technology), PKM2 (1:1000; Cell Signaling Technology), PKM1 (1:1000; Cell Signaling Technology), IGF2BP3 (1:5000, Abcam, Cambridge, MA, USA), and GAPDH (1:3000; Cell Signaling Technology). Secondary antibody HRP-labeled rabbit IgG (1:5000; Cell Signaling Technology) was probed for 1 h at room temperature. The bands were detected by enhanced chemiluminescence.

### Immunohistochemistry (IHC) and hematoxylin and eosin (HE) staining

Mouse tumor tissues were immobilized in 4% paraformaldehyde, embedded in paraffin and, cut into 4 μm sections. After deparaffinization, permeabilization, antigen retrieval, and incubation in 3% H_2_O_2_, tissue sections were incubated with primary antibodies PCNA (1:500, Abcam), PKM2 (1:800; Cell Signaling Technology), PKM1 (1:1000; Cell Signaling Technology), hnRNPA1 (1:100; Abcam). Then, the sections were probed with goat anti-rabbit biotinylated secondary antibody (1:3000, Abcam). Immunoreactive signals were visualized using DAB HRP Kit (Beyotime Biotechnology, Shanghai, China) and the nuclei were counterstained with hematoxylin (Sigma Aldrich).

Hematoxylin and eosin (HE) was performed to stain paraffin-embedded tissue sections. Sections were cut into 4 μm slices, and cytoplasm and nuclei were stained with eosin (Sigma Aldrich) and hematoxylin. The images were observed under a microscope.

### Cell counting kit-8 assay

GC cells were seeded at 1 × 10^4^ cells per well in 96-well plates, and cell viability was quantified according to the instructions of a Cell Counting Kit-8 assay (Sigma Aldrich) at 0 h, 24 h, 48 h, and 72 h. The optical density (OD) was detected at 450 nm through a Microplate Reader (Thermo Fischer Scientific, Waltham, MA, USA).

### 5-Ethynyl-2′-deoxyuridine (EdU) assay

Cell proliferation was measured with the EdU assay kit (Ribobio). Briefly, cells were treated with EdU for 2 h and then stained by 4′,6-diamidino-2-phenylindole (DAPI) (Thermo Fisher Scientific, CA, USA). The EdU-positive cells were visualized under a fluorescence microscope (Olympus, Tokyo, Japan).

### Colony formation

Transfected cells were inoculated in a 6-well plate at 200 cells per well. After incubation for 14 days, the colonies were fixed with formaldehyde and stained with 0.1% crystal violet (Sigma Aldrich). The number of visible colonies was calculated, and the images were captured under the microscope.

### Transwell invasion assay

Cell invasion ability was carried out using 24-transwell chambers with 8 μm pore size (Corning Incorporated, Corning, New York, USA) coated with Matrigel (Corning Incorporated). Transfected cells in serum-free medium were seeded in the upper chamber. The medium containing 10% FBS was added to the lower chamber. After being cultured for 24 h, cells that had invaded through the membrane were fixed with paraformaldehyde and stained with 0.1% crystal violet. Then cells were counted and photographed with the microscope.

### Wound-healing assay

Infected cells were seeded into 6-well plates at 1 × 10^5^ cells per well and cultured to a 90% confluence state. The cell was scratched by a 200 μ/l pipette tip. After washing with medium, the cells were incubated for 24 h. Images were captured at 0 and 24 h using a microscope and were analyzed using Image version 6.0 (Media Cybernetics, Rockville, MD, USA).

### RNA immunoprecipitation (RIP) assay

EZMagna RIP kit (Millipore, Bedford, MA, USA) was utilized to perform the RIP assay. In brief, transfected cells were lysed by lysis buffer, and the lysates were incubated with magnetic beads conjugated with anti-HNRNPA1, anti-IGF2BP3, or negative control IgG antibody. Proteinase K was treated to digest protein and RNAs from the beads. Finally, the immuno-precipitated RNA was determined by qRT-PCR.

### Co‑immunoprecipitation (Co-IP) assay

Co-IP experiments were performed using the Pierce Crosslink Magnetic IP/Co-IP Kit (Thermo Fisher Scientific). In brief, the protein A/G magnetic beads were pre-bound to anti-HNRNPA1, anti-Trim21, or IgG antibody and then incubated with cell lysates.

overnight at 4 °C. Subsequently, the antigens were dissociated from the antibody–bead complex with a low-pH elution buffer. The elution products were evaluated by western blot.

### RNA pull-down and truncation assays

Biotin-labeled sense, antisense, and m6A (GAACT) motif mutated OIP5-AS1 were synthesized using a T7 Transcription Kit (Thermo Fisher Scientific). RNA pull-down assay was performed to detect the proteins that bind to OIP5-AS1 using a Pierce Magnetic RNA–Protein Pull-Down Kit (Thermo Fisher Scientific). Cells were lysed using the lysis buffer. Cell lysates were incubated with streptavidin-coated magnetic beads for 3 h at 4 °C to get the biotin-coupled RNA complex. The proteins in RNA-binding protein complexes were identified by western blot analysis.

Truncation assay: The binding sequence of OIP5-AS1 to hnRNPA1 was divided into six 500 nucleotide (nt) segments: 0–2500 nt (F1), 0–500 nt (F2), 501–1000 nt (F3), 1001–1500 nt (F4), 1501–2000 nt (F5), and 2001–2500 nt (F6).

RNA pull-down was used to identify sequences that potentially bind to hnRNPA1 (biotin-labeled sequences, F1–F6).

### RNA stability assay

The expression of OIP5-AS1 in IGF2BP3- and METTL3-knockdown AGS cells was examined to explore whether IGF2BP3 affects OIP5-AS1 stability in GC cells. TRIzol reagent (Invitrogen) was used to extract total RNA from IGF2BP3- and METTL3-knockdown AGS cells treated with 5 mg/mL actinomycin D for 0, 3, 6, 9, or 12 h. The half-life of OIP5-AS1 was then determined as previously described [20].

### Ubiquitination assay

After 12 h of treatment with 25 mmol/L MG132 (Sigma Aldrich), the transfected cells were lysed using IP lysis buffer containing protease and phosphatase inhibitors. Subsequently, the same amount of lysate was incubated with anti-hnRNPA1 antibody overnight at 4 °C with rotation, followed by incubation with A/G magnetic beads at room temperature for 2 h with rotation. The A/G beads with RNA/protein mixture were collected, washed, and dissolved. The precipitated proteins were incubated with an anti-ubiquitin antibody (Cell Signaling Technology) [21].

### Fluorescence in situ hybridization (FISH)

OIP5-AS1, U6, and 18S FISH probes were purchased from RiboBio (Guangzhou, China), and the FISH kit was used to perform the FISH experiment (RiboBio). Cells fixed with 4% polyoxymethylene were incubated with 0.5% Triton X-100 solution at 4 °C for 5 min. Cells were washed thrice with PBS before being treated with pre-hybridization buffer at 37 °C for 30 min. After removing the pre-hybridization buffer, a 20 µM probe mix was added and incubated overnight at 37 °C. Cells were stained with DAPI for 10 min, sealed, and observed under a confocal microscope (Leica, Solms, Germany).

### Methylated RNA immunoprecipitation (MeRIP) assay

M6A modifications of OIP5-AS1 were evaluated using the Magna MeRIP™ m6A Kit (Merck, Darmstadt, Germany) according to the manufacturer’s recommendations. Briefly, TRIzol (Invitrogen) was used to isolate total RNA. RNA samples were immunoprecipitated with anti-m6A antibody or IgG overnight at 4 °C with rotation, followed by incubation with protein A/G beads overnight at 4 °C with rotation. Finally, the m6A-bound RNA was incubated with the MeRIP reaction mixture overnight at 4 °C. The OIP5-AS1 level in the m6A-bound RNA was detected by qRT-PCR.

### Glucose uptake and lactate assays

The supernatants of the transfected cells were harvested. Glucose and lactate concentrations were determined by glucose uptake and lactate assays using a Glucose Uptake-Glo™ Assay Kit (Promega, Madison, WI, USA) and a Lactate Colorimetric Assay Kit (Biovision, Milpitas, CA, USA), respectively, according to the manufacturer’s protocols.

### Subcellular RNA fractionations

qRT-PCR was performed to analyze nuclear and cytoplasmic RNA fractionations using the PARIS™ Kit (Invitrogen) according to the manufacturer’s instructions. 18 s and U6 were used as the cytoplasmic and nuclear endogenous controls, respectively.

### Extracellular acidification rate and oxygen consumption rate assays

The extracellular acidification rate (ECAR) and the cellular oxygen consumption rate (OCR) were determined using Seahorse XF Glycolysis Stress Test Kit (Agilent Technologies, Palo Alto, CA, USA) and Seahorse XF Cell Mito Stress Test Kit (Agilent Technologies) on Seahorse Extracellular Flux Analyzer XF24 (Seahorse Bioscience, MA, USA). Briefly, cells were seeded into an XFe24-well plate (8000 cells/well) for 48 h. After baseline measurements, for ECAR, 10 mM glucose, 1 µM oligomycin, and 50 mM 2-deoxy-D-glucose (2-DG) were automatically injected successively. For OCR, the seahorse automatically filled each well with 1 µM oligomycin and 1 µM p-trifluoromethoxy carbonyl cyanide phenylhydrazone (FCCP), respectively and 0.5 µM rotenone plus antimycin A (Rote/AA) successively. Seahorse XF24 Wave software was used to analyze the data of ECAR and OCR. ECAR in mpH/min and OCR in pmol/min are reported [22].

### Patient-derived xenograft model

Six-week-old healthy male NOD/SCID mice (weighing 20 ± 2 g) were purchased from the Laboratory Animal Center of Southern Medical University (Guangzhou, China) and housed in Affiliated Nanhua Hospital, Hengyang Medical School, University of South China standard experimental pathogen-free facility (25 °C, 50% humidity, 12-h light–dark cycle) with free access to water and food. All procedures were approved by the Affiliated Nanhua Hospital, Hengyang Medical School, University of South China Institutional Animal Care and Use Committee (No. 2021-KY-156). Pieces of the primary GC tissues from patients (approximately 50 mm^3^ each) were subcutaneously implanted into NOD/SCID mice. When the xenografted tumors grew up to 50 mm^3^, 18 mice were randomly divided into three groups (n = 6 mice /group): NC group (mice have systematically injected NC lentivirus through the lateral tail vein), sh-OIP5-AS1-1 group (mice have systematically injected sh-OIP5-AS1-1 lentivirus through the lateral tail vein), sh-OIP5-AS1-2 group (mice have systematically injected sh-OIP5-AS1-2 lentivirus through the lateral tail vein). Tumor volume was measured every four days, and mice were euthanized 40 days later. The weight of xenografts was determined, and the tumor volume was calculated using the formula *V* = 1/2 × width^2^ × length.

### In vivo animal model metastasis assay

A total of 18 6-week-old healthy male NOD/SCID mice (weighing 20 ± 2 g) were randomly assigned to one of the three groups (*n* = 6 mice /group) and injected through the tail vein with 1 × 10^6^ NC, sh-OIP5-AS1-1, or sh-OIP5-AS1-2 stable AGS cells or Ctrl or OIP5-AS1 stable MGC823 cells co-transfected with lentiviruses carrying the luciferase reporter gene. Mice were monitored using an IVIS imaging system (Caliper Life Sciences, Boston, MA, USA). Mice were sacrificed after eight weeks, and the lung tissues were collected for HE staining.

### Protein stability assay

The infected cells were harvested after being treated with 50 µg/ml of cycloheximide (CHX), a protein synthesis inhibitor, for the indicated times. hnRNPA1 protein expression was then evaluated by western blot analysis.

### Statistics analysis

Statistical analyses were performed using SPSS22.0 (SPSS Inc., Chicago, IL, USA) and GraphPad Prism 5.0 (GraphPad Software Inc., San Diego, CA, USA). The results were presented as mean ± standard deviation (SD). Differences between the two groups were evaluated using Student’s t-test. Differences among more than two groups were analyzed using one-way ANOVA. P < 0.05 was considered statistically significant.

## Results

### High expression of OIP5-AS1 was closely associated with the clinical parameters in GC patients

**Fig. 1:**
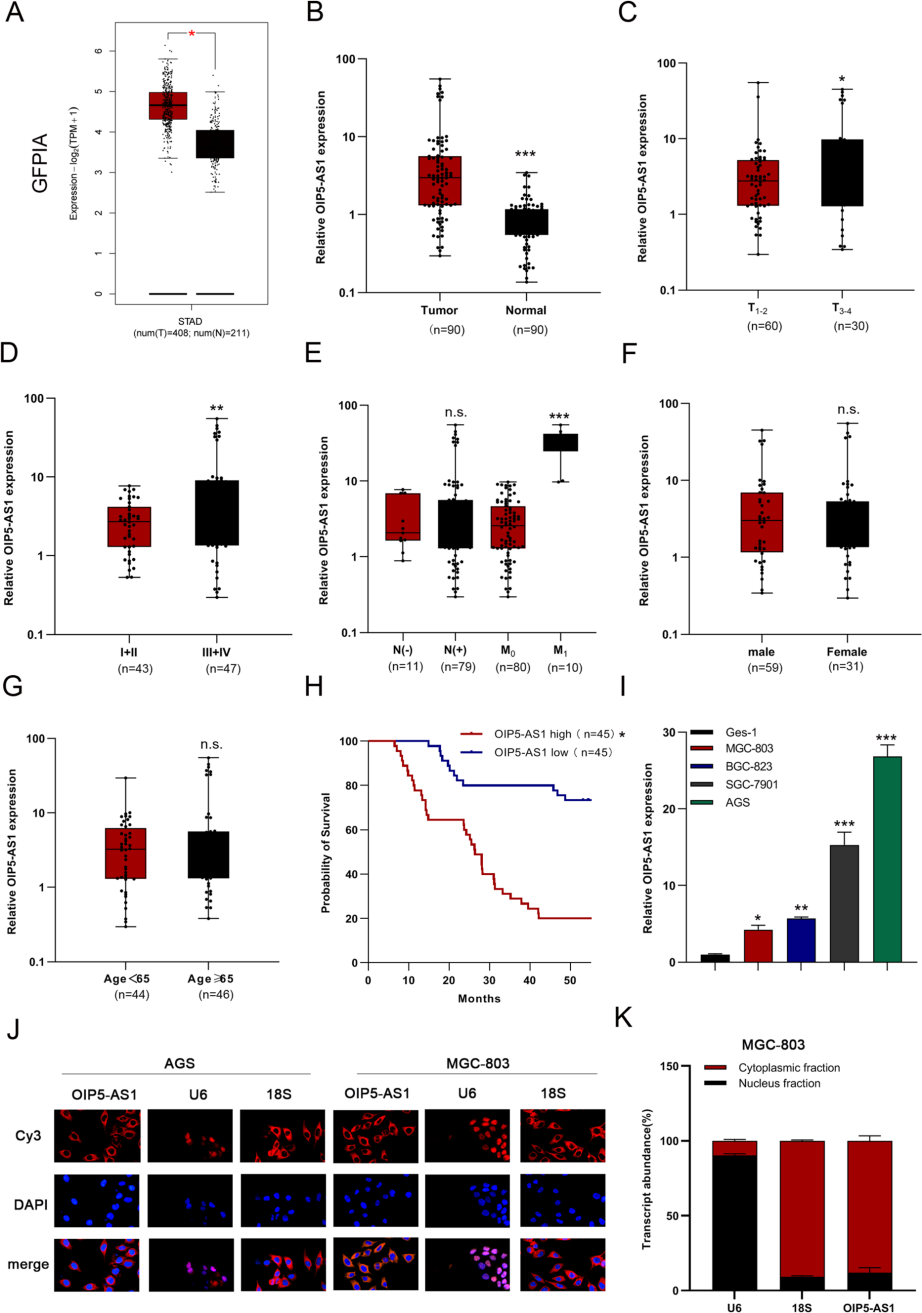
High expression levels of OIP5-AS1 were closely associated with the clinical parameters in GC patients. **A** OIP5-AS1 was overexpressed in GC tissues and paired with adjacent normal tissues in GEPIA STAD database analysis. **B** Relative expression of OIP5-AS1 in GC tissues and normal tissues was measured by qRT-PCR. **C–G** Correlation of OIP5-AS1 expression with age, sex, clinical stage, lymphatic metastasis, or distant metastasis. **H** Kaplan–Meier survival analysis with the log-rank test was used to analyze overall survival. **I** qRT-PCR was performed to detect the expression of OIP5-AS1 in GC cell lines. **J**, **K** FISH and subcellular fractionation analyses of the subcellular distribution of OIP5-AS1 in MGC-803 and AGS cells. The data in I–K are presented as the mean ± standard deviation (SD) (*n* = 3). *P < 0.05, **P < 0.01, ***P < 0.001

We initially analyzed the GEPIA STAD database and found that OIP5-AS1 levels were significantly increased in GC tissues compared with adjacent non-tumor tissues (Fig. 1A). We used qRT-PCR to detect OIP5-AS1 expression in 90 pairs of GC samples and corresponding noncancerous stomach samples. We found that OIP5-AS1 expression was higher in tumor tissues than in normal tissues (Fig. 1B). We then examined the relationship between OIP5-AS1 expression and clinicopathological parameters in GC patients and identified that OIP5-AS1 was positively and significantly correlated with the TNM stage, T stage, and distant metastasis (Figs. 1C–E). No significant differences were observed in age, sex, and lymphatic metastasis (Figs. 1E–G). Furthermore, Kaplan–Meier analysis demonstrated that patients with higher OIP5-AS1 expression levels had a worse overall survival rate than those with lower OIP5-AS1 expression levels (Fig. 1H). Consistent with the clinical results, OIP5-AS1 expression levels were significantly upregulated in GC cell lines (MGC-803, BGC-823, SGC-7901, and AGS) compared to the human normal gastric mucosa cell line GES-1 (Fig. 1I). Lastly, FISH and subcellular fractionation assays were used to verify that OIP5-AS1 was a cytoplasmic lncRNA in GC cells (Figs. 1J, K).

### OIP5-AS1 accelerates in vitro cell growth and colony formation and in vivo tumor formation

**Fig. 2:**
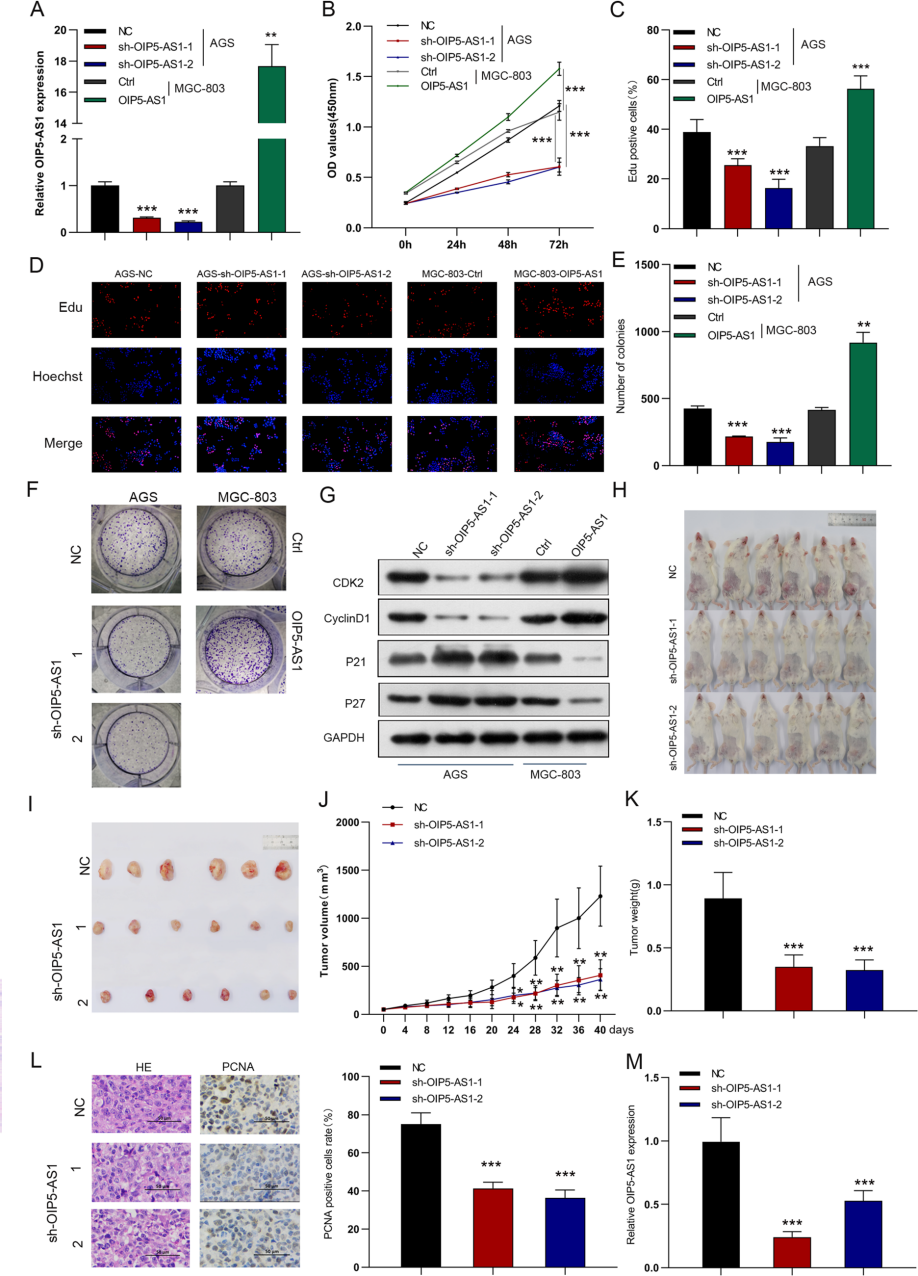
OIP5-AS1 accelerates cell growth and colony-formation in vitro and tumor formation in vivo. **A** The expression levels of OIP5-AS1 in AGS cells treated with sh-OIP5-AS1 or sh-NC lentiviruses and MGC-803 cells infected with OIP5-AS1 or control lentiviruses were detected by qRT-PCR. **B**–**F** Cell proliferation viability was analyzed by CCK-8, EdU, and colony-formation assays. **G** Effect of OIP5-AS1 on the expression level of cell proliferation-associated proteins. **H**–**K** The inhibitory effects of sh-OIP5-AS1 on tumor growth, volume, and weight. **L** IHC tested the expression of PCNA in tumors. **M** Relative expression of OIP5-AS1 in tumor tissues. The data in A–I are presented as the mean ± SD (*n* = 3). *P < 0.05, **P < 0.01, ***P < 0.001

To investigate the regulatory effects of OIP5-AS1 in GC cells, we used lentiviral vectors to overexpress OIP5-AS1 in MGC-803 cells stably and stably silence OIP5-AS1 in AGS cells using two distinct shRNA sequences (Fig. 2A). According to CCK-8 and EdU analysis, knocking out OIP5-AS1 dramatically attenuated cell proliferation ability, whereas overexpression of OIP5-AS1 facilitated cell proliferation (Figs. 2B–D). Similarly, OIP5-AS1 deficiency reduced the colony-formation ability of cells, whereas OIP5-AS1 overexpression promoted the growth of GC cells (Figs. 2E, F). Additionally, downregulation of OIP5-AS1 significantly decreased Cyclin D1 and CDK2 expression while increasing p21 and p27 expression, verified in OIP5-AS1 overexpressed cell lines (Fig. 2G). To verify the effect of OIP5-AS1 on GC tumor growth in vivo, patient-derived xenograft models were established using fresh GC tissues and inoculated with sh-OIP5-AS1 or shNC lentivirus in the tail vein. Both tumor volume and weight generated by the OIP5-AS1 silencing group were significantly lower than those generated by the control group (Figs. 2H–K). Moreover, IHC analysis revealed that the expression of the proliferation marker PCNA was reduced in the sh-OIP5-AS1-treated group (Fig. 2L). Also, the expression of OIP5-AS1 was markedly downregulated in the xenografts with blocked OIP5-AS1 compared to the control group (Fig. 2M).

### OIP5-AS1 promotes GC cell migration and invasion in vitro and the xenograft tumor metastasis in vivo

**Fig. 3:**
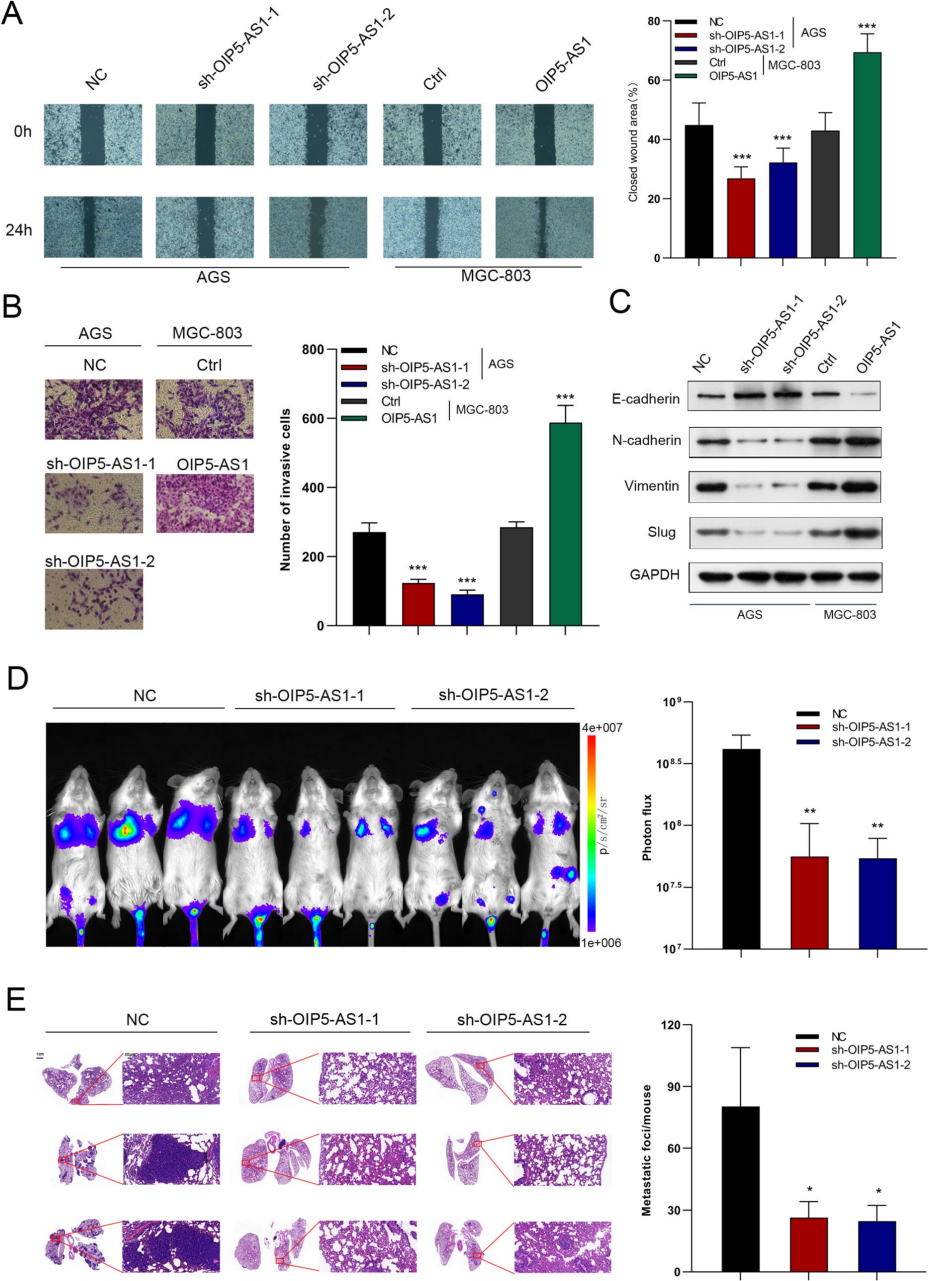
OIP5-AS1 promotes GC cell migration and invasion in vitro and xenograft tumor metastasis in vivo. **A**, **B** Cell migration and invasion ability were examined by wound-healing and transwell invasion assays in OIP5-AS1-silenced and OIP5-AS1-overexpressing GC cells, respectively. **C** The expression levels of EMT-associated proteins were evaluated by western blot. **D** Representative images of lung metastasis by a live imaging system and quantification of photon flux for metastases. **E** Representative images and quantification of lung metastasis by HE staining. The data in A–C are presented as the mean ± SD (*n* = 3). *P < 0.05, **P < 0.01, ***P < 0.001

Following that, we performed wound-healing and transwell invasion assays to investigate the functions of OIP5-AS1 in the metastatic behavior of GC cells. As shown in Fig. 3A, silenced OIP5-AS1 expression was significantly inhibited, whereas forced OIP5-AS1 expression enhanced the migration capacity of GC cells. Similarly, OIP5-AS1-silenced cells invaded substantially slower, whereas OIP5-AS1-overexpressed cells invaded more quickly than corresponding control cells (Fig. 3B). OIP5-AS1 silencing upregulated epithelial–mesenchymal transition (EMT)-associated protein E-cadherin expression and down-regulated N-cadherin, vimentin, and slug expression, but OIP5-AS1 overexpression had the opposite effect, as determined by western blot (Fig. 3C). To investigate the role of OIP5-AS1 in tumor metastasis in vivo, researchers injected luciferase-labeled Ctrl-, OIP5-AS1-, sh-OIP5-AS1-, or shNC-transfected cells into the tail veins of NOD-SCID mice. Figs. 3D, E Supplementary Figs. 1A, B shows a significant reduction in metastatic outgrowth in the lungs of OIP5-AS1-silenced group mice, while a significant increase in metastatic outgrowth in the lungs of OIP5-AS1-overexpression group mice.

### OIP5-AS1 regulates the Warburg effect in GC cells

**Fig. 4:**
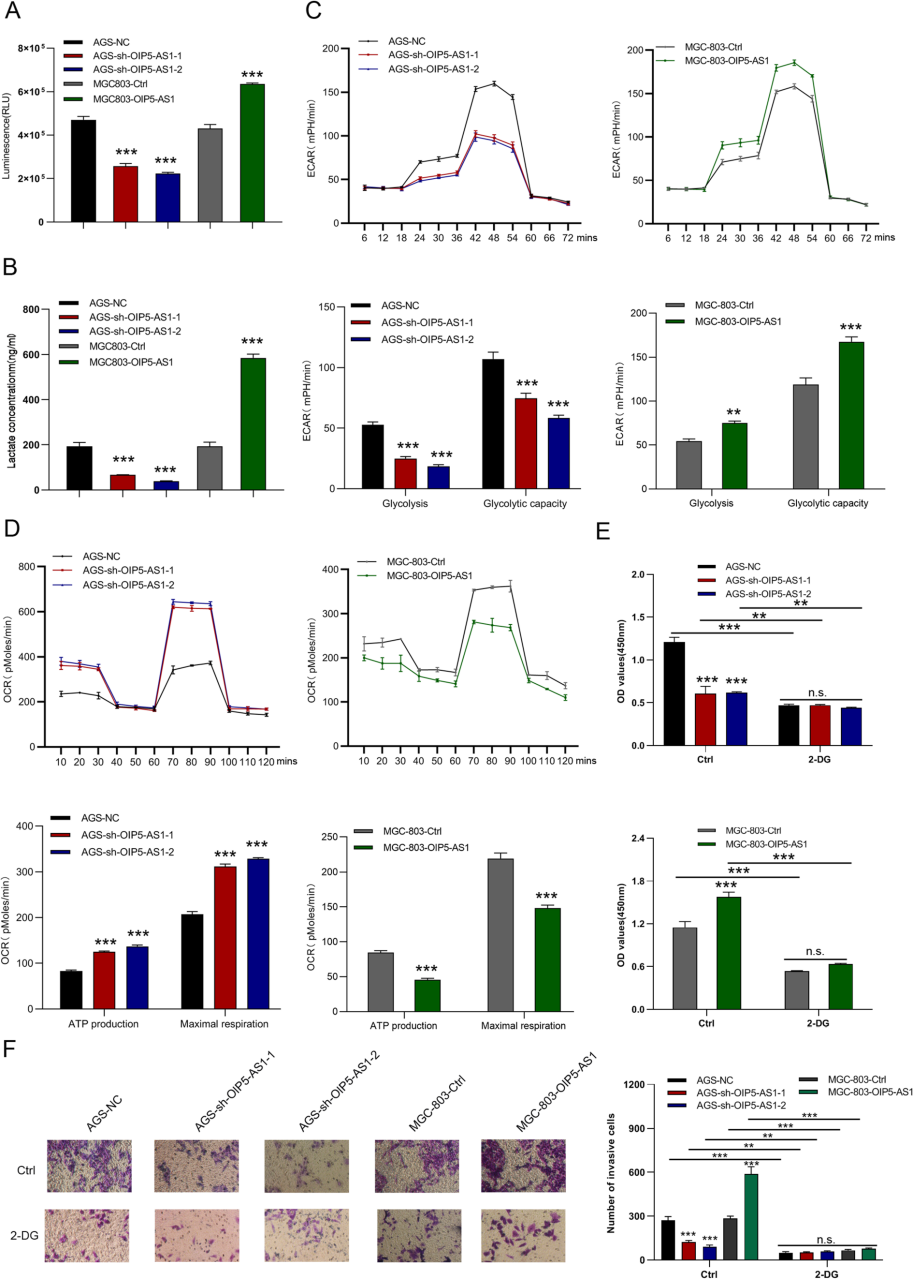
OIP5-AS1 regulates the Warburg effect in GC cells. **A**, **B** Effects of OIP5-AS1 knockdown and overexpression on glucose consumption and lactate production **C** ECAR analysis of OIP5-AS1-depleted AGS cells and OIP5-AS1-overexpressing MGC-803 cells were performed with the Seahorse XF Glycolysis Stress Test Kit. **D** Diagram and quantitative analysis of OCR measurement with Seahorse XF Cell Mito Stress Test Kit. **E**, **F** The proliferation and metastasis status of 2-deoxyglucose-treated OIP5-AS1-depleted AGS cells and OIP5-AS1-overexpressed MGC-803 cells. All data are presented as the mean ± SD (*n* = 3). **P < 0.01, ***P < 0.001

Aerobic glycolysis up-regulation can promote the proliferation and metastasis of cancer cells [23]. Therefore, we performed glucose uptake, lactate, and seahorse assays to examine whether OIP5-AS1 affects aerobic glycolysis. As expected, cells with OIP5-AS1 silencing showed a significant decrease in glucose uptake and lactate production. However, cells overexpressing OIP5-AS1 increased glucose uptake and lactate production (Figs. 4A, B). Additionally, knockdown of OIP5-AS1 inhibited glycolysis and glycolytic capacity, whereas OIP5-AS1 overexpression significantly enhanced the glycolysis and glycolytic capacity of GC cells (Fig. 4C). OIP5-AS1 knockdown markedly increased ATP production and maximal respiration, whereas these factors were reduced by forced OIP5-AS1 expression (Fig. 4D). The glycolytic inhibitor, 2-deoxyglucose (2-DG), was then used to evaluate whether OIP5-AS1 affects cell proliferation and metastasis by regulating aerobic glycolysis. Treatment with 2-DG suppressed the proliferation and metastasis of OIP5-AS1-depleted and OIP5-AS1-overexpressing cells in CCK-8 and Transwell invasion assays (Figs. 4E, F).

### IGF2BP3 regulates OIP5-AS1 expression in an m6A-dependent manner

**Fig. 5:**
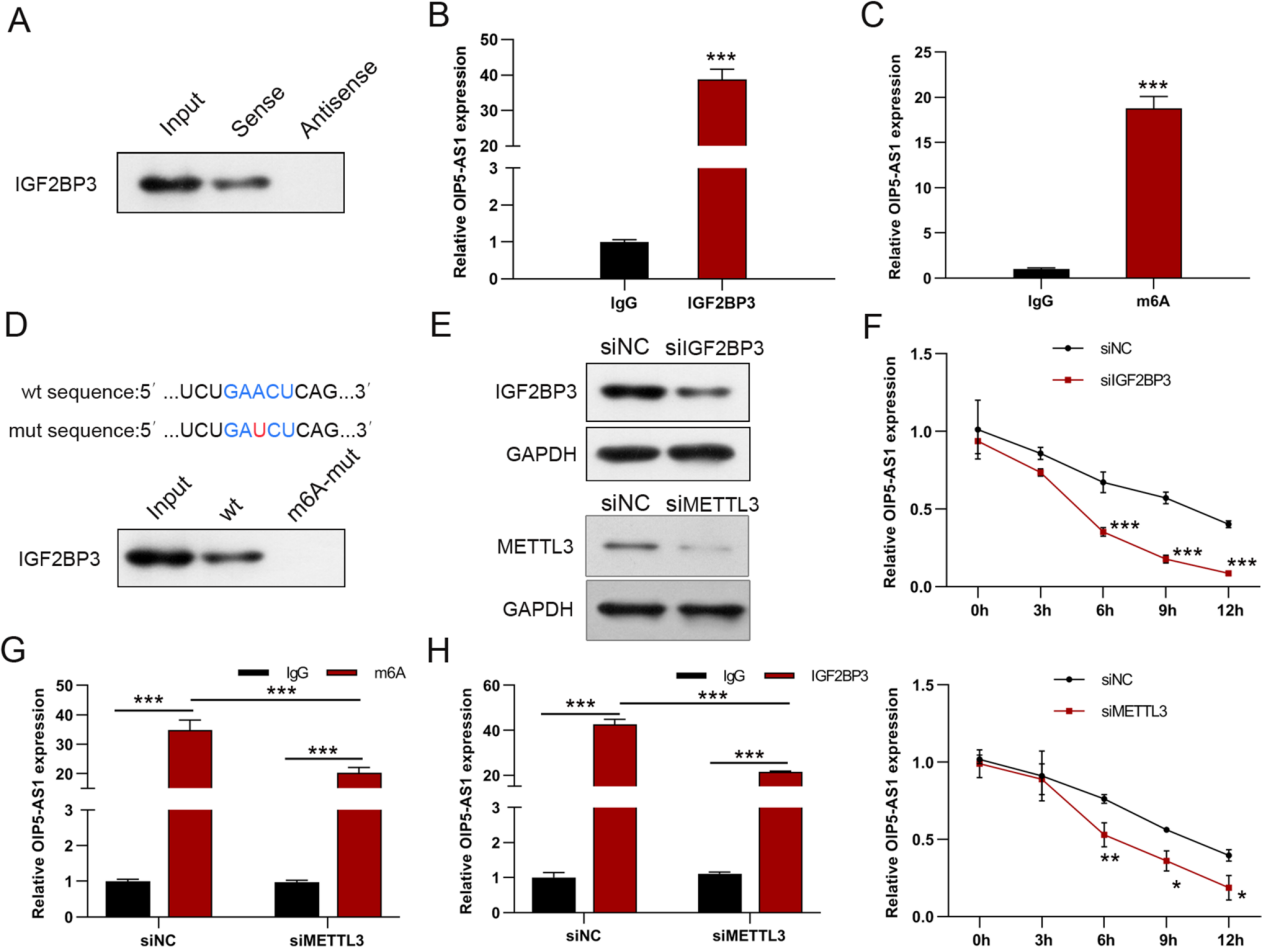
IGF2BP3 regulated OIP5-AS1 expression in an m6A-dependent manner. **A**, **B** RNA pull-down and RIP assays confirmed the interaction between IGF2BP3 and OIP5-AS1. **C** The obvious m6A modification of OIP5-AS1 was confirmed by MeRIP. **D** Top: Schematic display of the m6A motif (GAACU) in OIP5-AS1 and mutation used in the subsequent RNA pull-down assay. Down: The OIP5-AS1 mutation of the m6A-binding motif reduced the binding of IGF2BP3 to OIP5-AS1. **E** The protein levels of IGF2BP3 in IGF2BP3-knockdown cells and METTL3 protein levels in METTL3-silenced cells. **F** The OIP5-AS1 stability and halftime in IGF2BP3- or METTL3-knockdown cells treated by Actinomycin D. **G**, **H** OIP5-AS1 enrichment in METTL3-silenced cells was detected by RIP assay (using IGF2BP2-specific antibody) or MeRIP assay (using m6A-specific antibody). All data are presented as the mean ± SD (*n* = 3). *P < 0.05, **P < 0.01, ***P < 0.001

To investigate the underlying mechanisms of OIP5-AS1 in the development of GC, starbase, RMBase V2.0, RM2Target and POSTAR3 were used to predict the targets of OIP5-AS1, and found that IGF2BP3 and YTHDC1 that bind to OIP5-AS1 (Supplementary Fig. 2A). According to GEPIA database and Kaplan–Meier Plotter analysis, only IGF2BP3 is upregulated in GC tissues and correlated with poor prognosis (Supplementary Figs. 2B-D). Meanwhile, a positive correlation between IGF2BP3 and OIP5-AS1 expression was observed in TCGA database (Supplementary Fig. 2E). Furthermore, IGF2BP3 is directly bound to OIP5-AS1 in AGS cells, as shown in RNA pull-down assays (Fig. 5A). Subsequent RIP assay validated the enrichment of OIP5-AS1 by IGF2BP3 antibody in AGS cells, which confirmed the interaction between IGF2BP3 and OIP5-AS1 (Fig. 5B). IGF2BP3, an m6A reader, promotes RNA stability in an m6a-dependent manner [20]. SRAMP (an m6A modification site predictor) was used to predict a classical m6A motif (GAACT) in the sequence of OIP5-AS1, so we hypothesized that OIP5-AS1 might exhibit m6A modification in AGS cells. MeRIP assay was performed to validate the m6A modification in OIP5-AS1 (Fig. 5C). Moreover, mutating the m6A motif in OIP5-AS1 exhibited a significant decline in the binding of IGF2BP3, as shown by RNA pull-down assays, suggesting that the binding of IGF2BP3 to OIP5-AS1 depends on m6A modification (Fig. 5D). METTL3, an essential m6A methyltransferase known as an m6A writer, is highly expressed in GC tissues, associated with poor prognosis of patients, and positively linked to OIP5-AS1 by TCGA data and Kaplan–Meier Plotter analysis (Supplementary Figs. 2F-H). Thus, we knocked down IGF2BP3 or METTL3 in GC cells to investigate whether IGF2BP3 regulated OIP5-AS1 stability, and found that IGF2BP3 expression was significantly reduced (Fig. 5E). We then investigated OIP5-AS1 stability over time using the transcription inhibitor Actinomycin D treatment. The silencing of IGF2BP3 led to an obvious decrease in stability and halftime of OIP5-AS1 (Fig. 5F). Similarly, the loss of METTL3 markedly shortened the halftime of OIP5-AS1 (Fig. 5F). METTL3 knockdown significantly affected the enrichment of OIP5-AS1 in RIP assays when using either IGF2BP3- or m6A-specific antibody compared to the controls (Figs. 5G, H).

### IGF2BP3 regulates the bioactivity of GC cells through OIP5-AS1

**Fig. 6:**
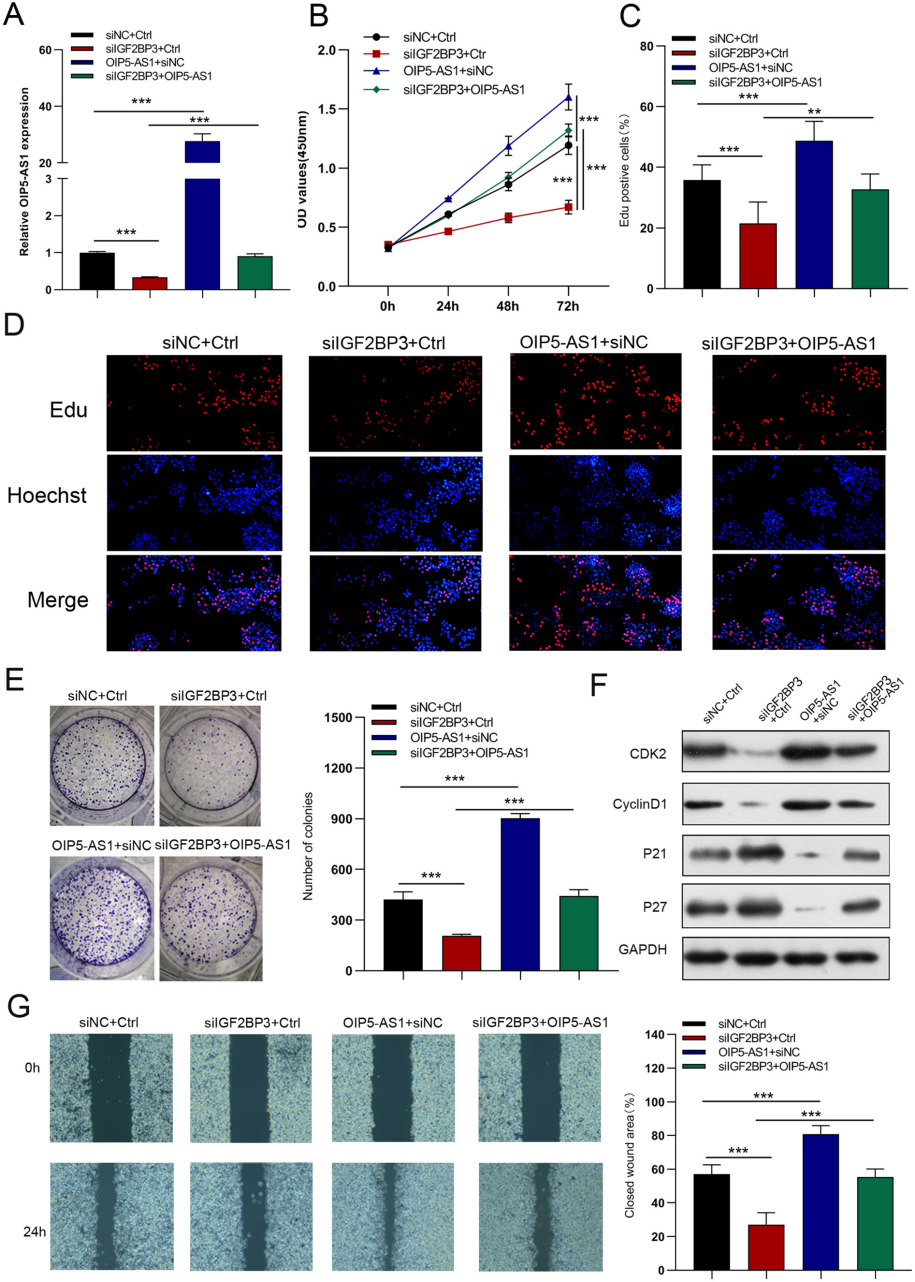
IGF2BP3 regulates the bioactivity of GC cells through OIP5-AS1. **A** The relative expression of OIP5-AS1 was examined by qRT-PCR analysis in GC cells co-transfected with siIGF2BP3 plus OIP5-AS1 lentiviruses. **B**–**G** Cell growth, migration, and invasion were detected by CCK-8, EdU, colony-formation, and wound-healing assays

**Fig. 7:**
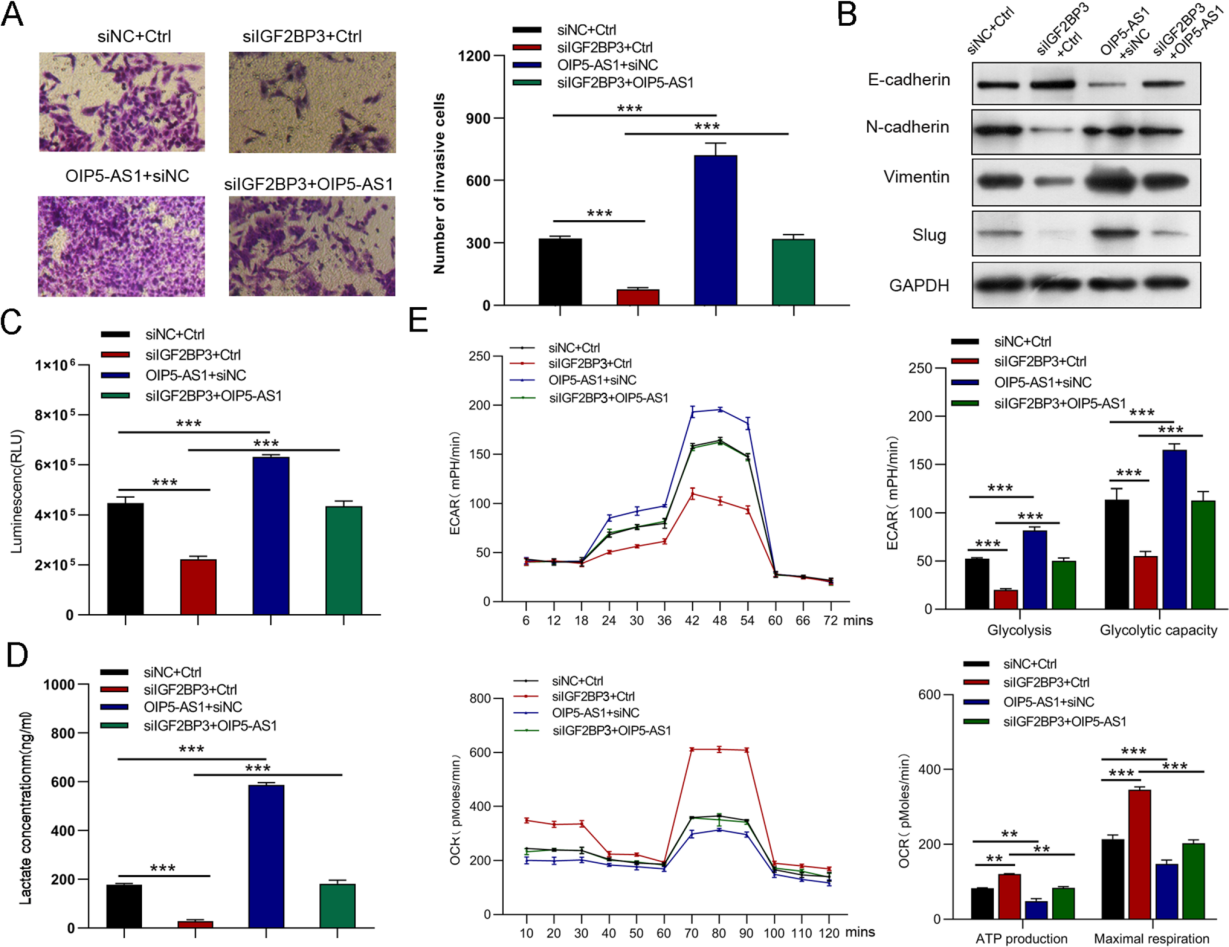
IGF2BP3 regulates GC cell invasion, EMT, and glucose metabolism through OIP5-AS1. **A**, **B** Cell invasion and EMT were measured via transwell and western blot assays. **C**–**E** Glucose uptake, lactate production, ECAR, and OCR in GC cells. All data are presented as the mean ± SD (*n* = 3). **P < 0.01, ***P < 0.001

AGS cells were co-transfected with siIGF2BP3 and OIP5-AS1 lentiviruses to investigate whether IGF2BP3 is involved in the OIP5-AS1 mediated biological function of GC cells. qRT-PCR results indicated that the expression levels of OIP5-AS1 in siIGF2BP3-transfected cells were partially increased by OIP5-AS1 overexpression in GC cells (Fig. 6A). IGF2BP3 knockdown significantly inhibited the cell proliferation, migration, and invasion of GC cells, whereas these effects were rescued by OIP5-AS1 upregulation (Figs. 6B–G, 7A, B). Additionally, decreased glucose uptake, lactate production, glycolysis, glycolytic capacity, increased ATP production, and maximal respiration in IGF2BP3-silenced cells were reversed by overexpression of OIP5-AS1 (Figs. 7C–E). These results suggested that the OIP5-AS1 inhibitor partially mediated the oncogene effect of IGF2BP3.

### OIP5-AS1 enhances hnRNPA1 stability by blocking Trim21-mediated ubiquitination and degradation of HNRNPA1

**Fig. 8:**
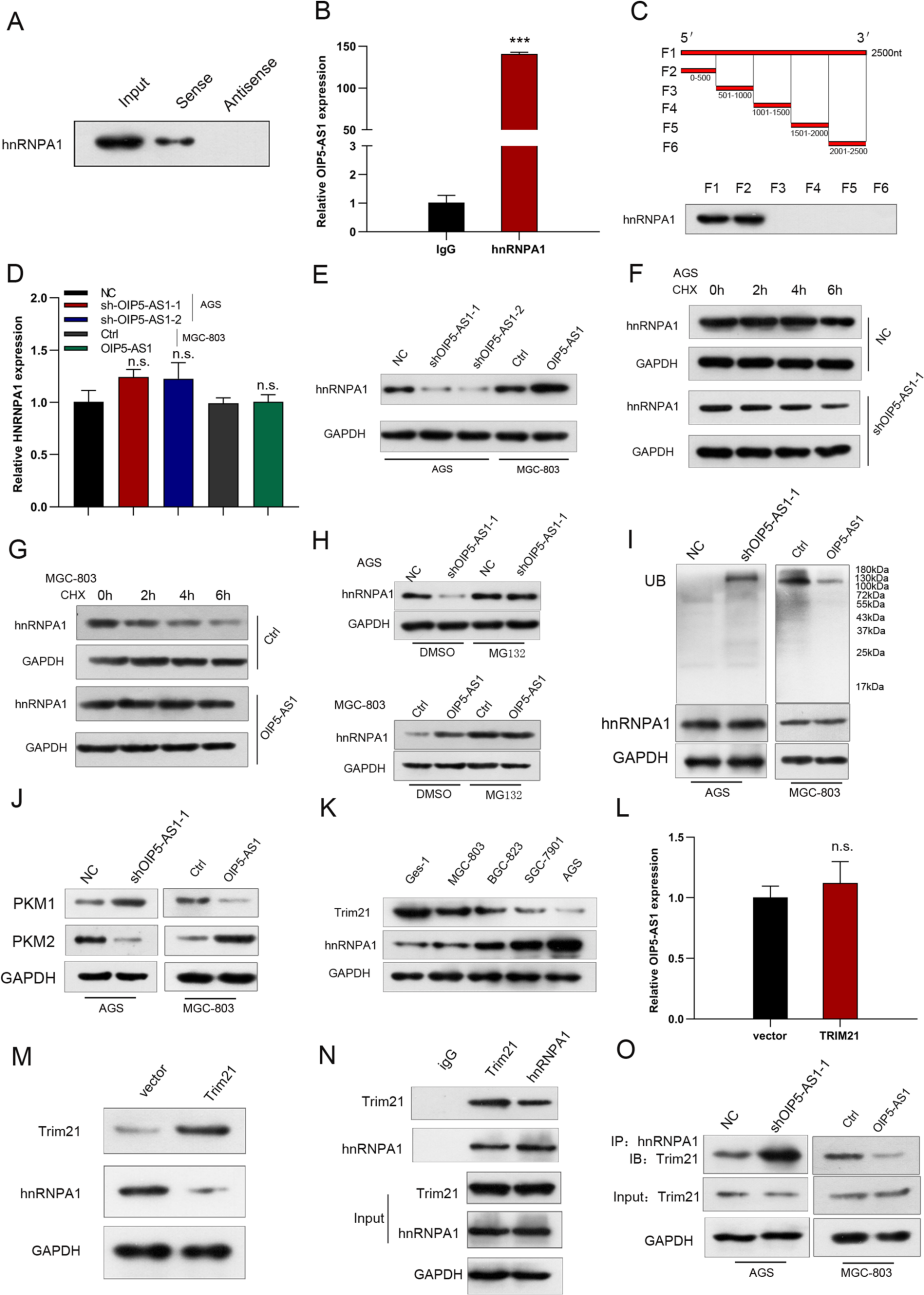
OIP5-AS1 enhances hnRNPA1 stability by blocking Trim21-mediated ubiquitination and degradation of HNRNPA1. **A**, **B** RNA pull-down and RIP assays were conducted to identify the interaction between OIP5-AS1 and HNRNPA1. **C** Truncation assays identified the OIP5-AS1 segment that binds to HNRNPA1. **D**, **E** The mRNA and protein expression levels in OIP5-AS1-knockdown and overexpressing cells were examined by qRT-PCR and western bolt, respectively. **F**–**H** The protein expression of hnRNPA1 in the OIP5-AS1- and sh-OIP5-AS1-transfected cells treated with cycloheximide (CHX, 50 µg/mL) for the indicated times or MG132 (25 mmol/L) for 12 h. **I** Western blot analysis of ubiquitin immuno-precipitated with anti-hnRNPA1 antibodies in the OIP5-AS1- and sh-OIP5-AS1-transfected cells treated with MG132 (25 mmol/L) for 12 h. **J** The protein expression levels of PKM1/2 in the OIP5-AS1- and sh-OIP5-AS1-transfected cells. **K** The protein expression levels of Trim21 and hnRNPA1 in GC cell lines and GES-1 cells. **L**, **M** Trim21, HNRNPA1, and OIP5-AS1 in AGS cells transfected with Trim21 plasmids. **N** CO-IP analysis revealed the Trim21/hnRNPA1 interaction in AGS cells. **O** CO-IP was used to assess the interaction between Trim21 and hnRNPA1 after OIP5-AS1 upregulation and downregulation in GC cells. All data are presented as the mean ± SD (*n* = 3)

Two candidate targets (hnRNPA1 and YTHDC1) of OIP5-AS1 were predicted using the bioinformatics software starbase, RBPDB, and POSTAR3 (Supplementary Fig. 2I). TCGA database and Kaplan–Meier Plotter analysis revealed that only hnRNPA1 is significantly overexpressed in GC tissues, and associated with poor overall survival (Supplementary Figs. 2J, K). There is a significant positive correlation between hnRNPA1 and OIP5-AS1 expression in TCGA database (Supplementary Fig. 2L). Furthermore, OIP5-AS1 knockdown also reduced the protein expression of hnRNPA1 in patient-derived xenograft models (Supplementary Fig. 1C). Next, we performed RNA pull-down and RIP assays to confirm OIP5-AS1 might interact with hnRNPA1 (Figs. 8A, B). According to truncation assays, hnRNPA1 binds to the 0–500 nt segment of OIP5-AS1 (Fig. 8C). The expression levels of hnRNPA1 in OIP5-AS1-knockdown AGS cells and OIP5-AS1-overexpressing MGC-803 cells were examined to clarify further the molecular mechanism of the interaction between OIP5-AS1 and HNRNPA1. We observed that hnRNPA1 mRNA levels remained unchanged in OIP5-AS1-silenced and OIP5-AS1-overexpressed cells (Fig. 8D). However, hnRNPA1 protein levels were significantly reduced in OIP5-AS1-downregulated cells and increased in OIP5-AS1-upregulated cells (Fig. 8E). Thus, we hypothesized that OIP5-AS1 modulates hnRNPA1 by post-transcriptional regulation. Ubiquitination degradation has been reported to be one of the most important molecular mechanisms of post-transcriptional regulation [21]. Protein stability assay revealed that knocking down OIP5-AS1 surprisingly shortened the half-life of the hnRNPA1 protein in GC cells, while overexpressed OIP5-AS1 dramatically increased hnRNPA1 protein half-life (Figs. 8F, G). Moreover, a decrease in the hnRNPA1 protein levels after OIP5-AS1 silencing was attenuated when GC cells were treated with the proteasome inhibitor MG132, whereas OIP5-AS1 overexpression has an opposite effect (Fig. 8H). Additionally, OIP5-AS1-knockdown cells had increased hnRNPA1 ubiquitination levels, but OIP5-AS1-overexpressing cells displayed decreased hnRNPA1 ubiquitination levels (Fig. 8I), indicating that OIP5-AS1 is involved in the proteasome-dependent degradation of hnRNPA1 in GC cells. It has been reported that hnRNPA1 influences energy metabolism via modulating the PKM alternative splicing [21]. PKM2 is increased in GC tissues, and patients with higher PKM2 expression suffered much more awful overall survival in GEPIA database and Kaplan–Meier Plotter analysis (Supplementary Figs. 2M, N). Also, PKM2 is positively associated with OIP5-AS1 in TCGA database (Supplementary Fig. 2O). In patient-derived xenograft models, inhibition of OIP5-AS1 significantly downregulated the expression of PKM2, and upregulated PKM1 expression (Supplementary Fig. 1C). Moreover, OIP5-AS1 knockdown enhanced PKM1 expression while reducing PKM2 expression (Fig. 8J). On the contrary, forced expression of OIP5-AS1 decreased the expression of PKM1, and increased PKM2 expression (Fig. 8J). Then, we used the computational predictive system UbiBrowser to verify the characteristics of the E3 ligases interacting with hnRNPA1 and found that Trim21 is an E3 ligase for HNRNPA1, and has been reported to be decreased in GC tissues and its down-regulation was correlated with lower overall survival rate among GC patients[24]. As shown in Fig. 8K, low levels of Trim21 and high levels of hnRNPA1 are observed in GC cell lines when compared to GES-1 cells. Furthermore, hnRNPA1 expression levels were downregulated in Trim21-overexpressing cells, although there was no significant difference in OIP5-AS1 expression levels (Figs. 8L, M). CO-IP assays were then performed to determine which Trim21 interacts with HNRNPA1, and we observed that Trim21 could bind to hnRNPA1 in AGS cells (Fig. 8N). Moreover, we examined the association between Trim21 and hnRNPA1 when OIP5-AS1 was silenced or overexpressed. Downregulation of OIP5-AS1 significantly improved, whereas upregulation of OIP5-AS1 reduced the interaction of Trim21 and hnRNPA1 (Fig. 8O).

### hnRNPA1 mediates the regulation of OIP5-AS1 in GC cells

**Fig. 9:**
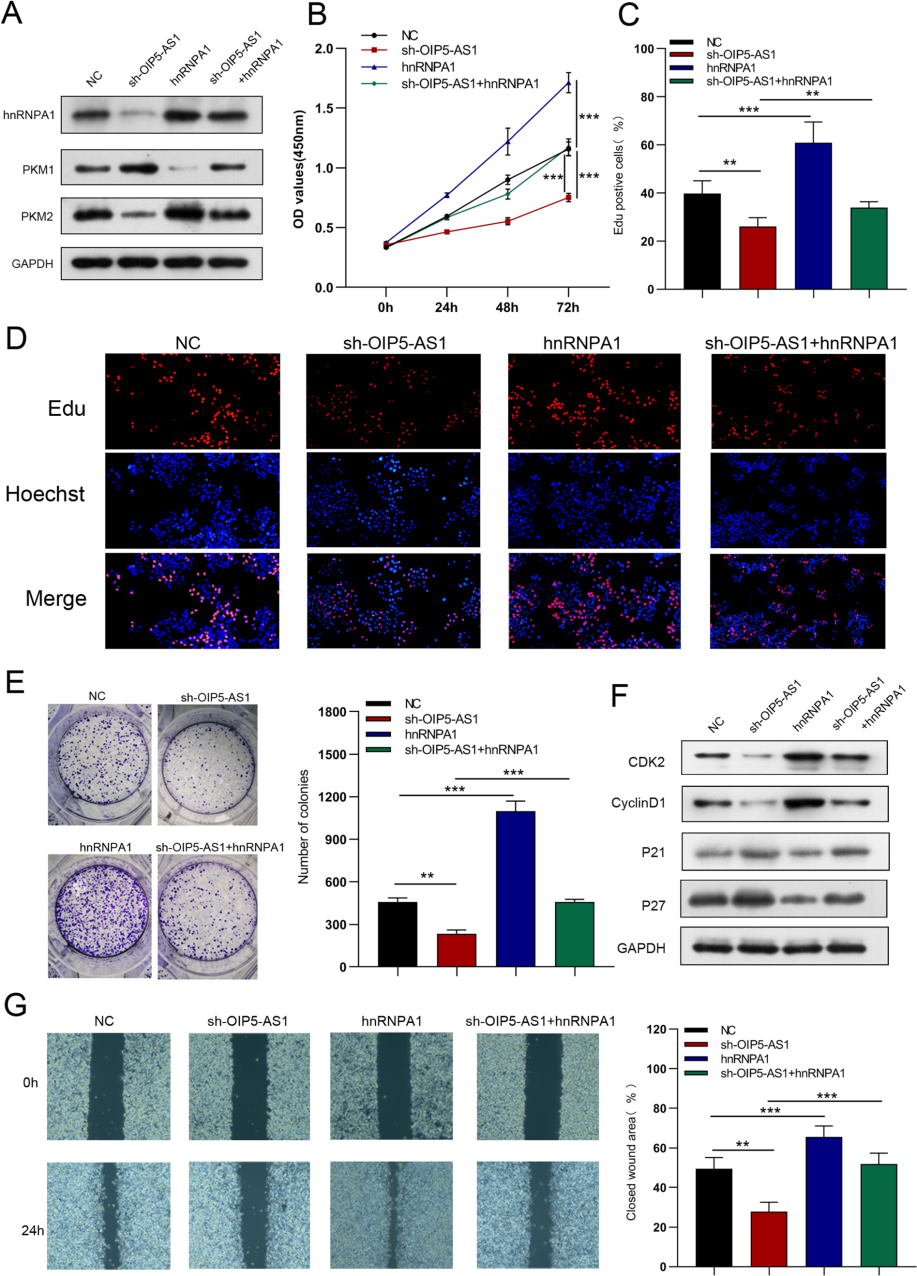
hnRNPA1 mediated the regulation of OIP5-AS1 in GC cells. **A** Western blot analysis on hnRNPA1, PKM1 and PKM2 expression in shOIP5-AS1-infected AGS cells transfected with hnRNPA1 plasmid. **B**–**G** CCK-8 assay, EdU, colony-formation, and wound-healing assays were performed to measure cell growth, and migration. All data are presented as the mean ± SD (*n* = 3). **P < 0.01, ***P < 0.001

**Fig. 10:**
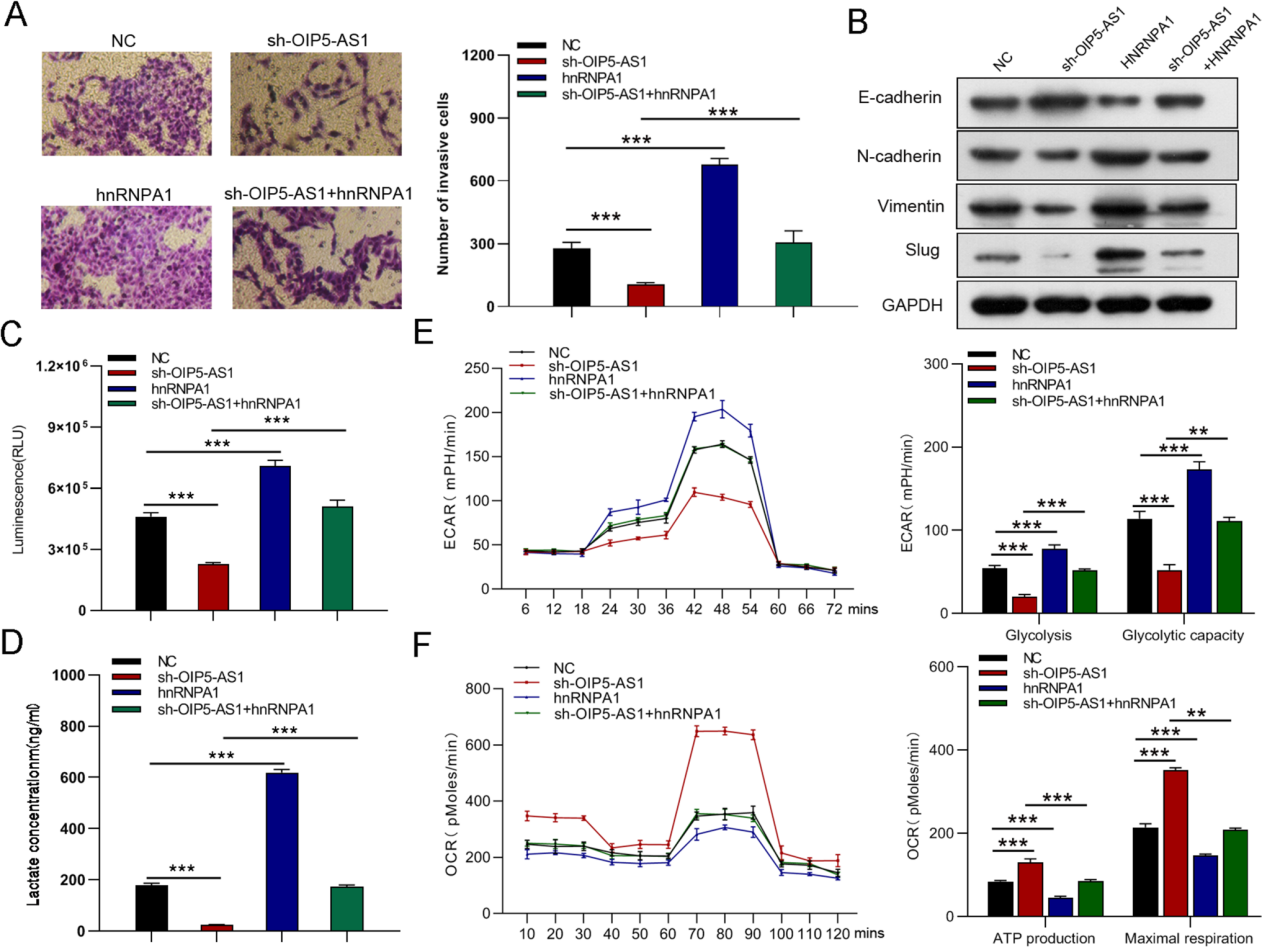
hnRNPA1 mediated the regulation of OIP5-AS1 on GC cell invasion, EMT and glycometabolism. **A**, **B** Transwell and western blot assays were performed to measure cell invasion and EMT. **C**–**F** Glucose uptake, lactate production, ECAR, and OCR analysis were used to determine glucose uptake, lactate production, glycolysis, glycolytic capacity, ATP production, and maximal respiration. All data are presented as the mean ± SD (*n* = 3). **P < 0.01, ***P < 0.001

**Fig. 11:**
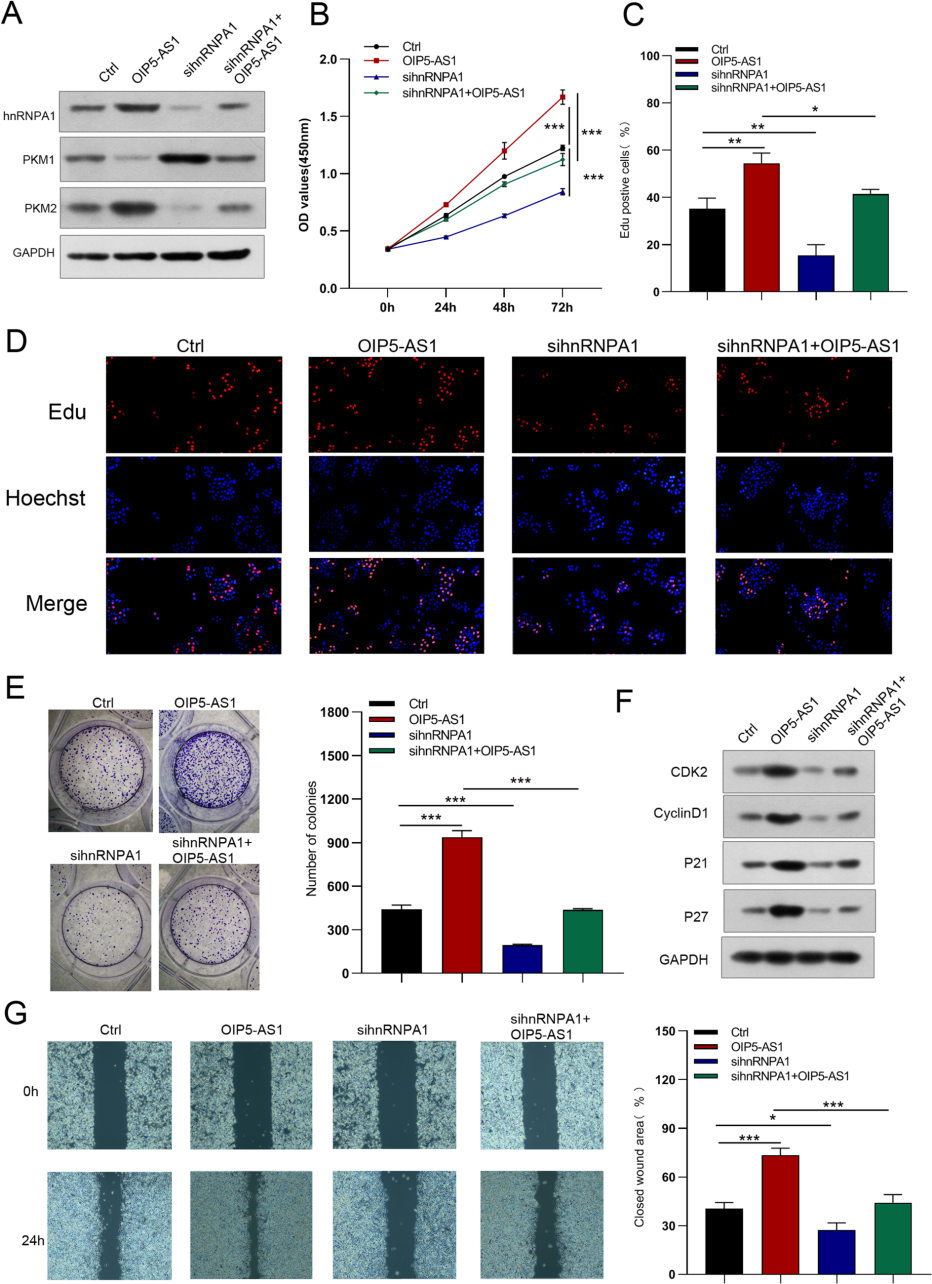
hnRNPA1 silencing abolished the facilitative effects of OIP5-AS1 on the growth and migration of GC cells. **A** Protein expression of hnRNPA1, PKM1 and PKM2 in OIP5-AS1-infected MGC823 cells transfected with hnRNPA1 siRNA. **B**–**G** CCK-8 assay, EdU, colony-formation, and wound-healing assays were used to detect the growth and migration of GC cells. All data are presented as the mean ± SD (*n* = 3). *P < 0.05, **P < 0.01, ***P < 0.001

**Fig. 12:**
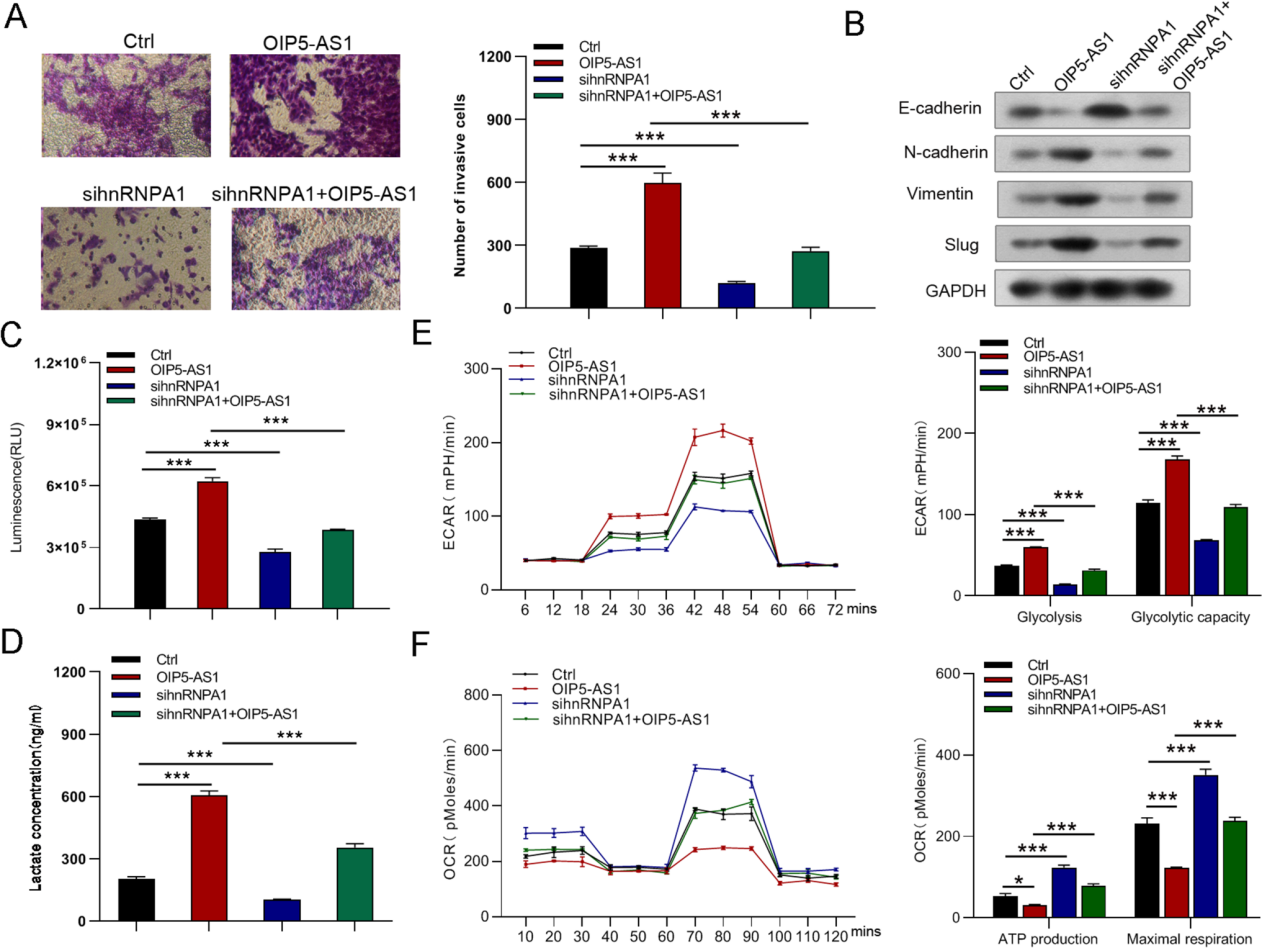
Knockdown of hnRNPA1 reversed the boosted the invasion, EMT and glycolysis of MGC823 cells induced by overexpression of OIP5-AS1. **A**, **B** Cell invasion and EMT were examined by Transwell and western blot assays, respectively. **C**–**F** Glucose uptake, lactate production, glycolysis, glycolytic capacity, ATP production, and maximal respiration in the different groups. All data are presented as the mean ± SD (*n* = 3). *P < 0.05, ***P < 0.001

To validate whether OIP5-AS1 could affect GC cell function that is regulated by HNRNPA1, we performed the rescue assays. As western blot analysis revealed that downregulated expression of hnRNPA1 and PKM2 protein and upregulated expression of PKM1 protein by sh-OIP5-AS1 could be reversed by overexpression of hnRNPA1 (Fig. 9A), but increased hnRNPA1 and PKM2 protein expression and decreased PKM1 protein levels, which was induced by OIP5-AS1 overexpression, were abrogated following hnRNPA1 silencing (Fig. 11A). Forced expression of hnRNPA1 partially rescued the OIP5-AS1 knockdown-mediated inhibition of GC cell growth, migration, and invasion (Figs. 9B–G, 10A, B) whereas inhibition of hnRNPA1 partially reversed the GC cell growth and metastasis promoting effects induced by OIP5-AS1 upregulation (Figs. 11B–G, 12A, B). Moreover, hnRNPA1 stimulation restored the reduced glucose uptake, lactate production, glycolysis, and glycolytic capacity, while enhancing ATP production and maximal respiration caused by OIP5-AS1 silence (Figs. 10C–F). However, hnRNPA1 downregulation eliminated the elevated glucose uptake, lactate production, glycolysis, and glycolytic capacity, and declined ATP production and maximal respiration induced by overexpression of OIP5-AS1 (Figs. 12C–F). In conclusion, OIP5-AS1 exhibited carcinogenesis activating hnRNPA1 by competitive binding to OIP5-AS1.

## Discussion

LncRNA’s abnormal expression or dysfunction has been linked to various biological and pathological processes and has been verified to play multiple roles in the occurrence and development of cancer [7]. OIP5-AS1 has been reported to have an oncogenic property in various malignant tumors, including hemangioma, hepatocellular carcinoma, bladder cancer, glioma, and GC [25–29]. Functional roles and molecular mechanisms of OIP5-AS1 in GC are still unknown. In this study, we observed that OIP5-AS1 was increased in GC tissues and cell lines and associated with the malignant features and prognosis of GC. Knockdown of OIP5-AS1 significantly inhibited cell proliferation, migration, invasion, and glucose metabolism in vitro and tumorigenicity and metastasis in vivo. In a nutshell, it has been validated that OIP5-AS1 functions as an oncogene in GC development.

We found that IGF2BP3 bound to the OIP5-AS1 using starbase, RMBase V2.0, RM2Target, and POSTAR3 analysis, which was confirmed by RNA pull-down and RIP assays. IGF2BP3, a member of the IGF2BP family, has been reported to interact with various lncRNAs to regulate many basic biological functions [30]. IGF2BP3 is an m6A reader essential in various malignancies [31]. The aberrantly high expression of IGF2BP3 can preferentially detect m6A-modified target RNAs, thereby facilitating target RNA stability and expression, causing tumor progression, angiogenesis, glycolysis, and chemoresistance [32–34]. In the present study, the presence of m6A sites in the OIP5-AS1 sequence was predicted by the RNA modification online database SRAMP. The MeRIP assay was conducted to identify the enrichment of m6A modification in the OIP5-AS1 sequence. The RNA pull-down assay further verified that the mutation of the m6A motif (GAACU) in OIP5-AS1 remarkably reduced the IGF2BP3 expression level. Moreover, IGF2BP3 and METTL3 increased the stability of OIP5-AS1 in GC cells treated with actinomycin D. Final rescue assays were performed to validate the ability of OIP5-AS1 to offset the inhibitory effect of IGF2BP3 knockdown on growth, migration, and invasion of GC cells. Therefore, IGF2BP3 may be an m6A modification regulator of OIP5-AS1 in GC.

LncRNAs can act in combination with different proteins to perform various functions depending on their subcellular location [35]. This study demonstrated that OIP5-AS1 is predominantly found in the cytoplasm. Based on the prediction of bioinformatics software starbase, RBPDB, and POSTAR3, we found that hnRNPA1 is one of the proteins bound to OIP5-AS1. An interaction between OIP5-AS1 and hnRNPA1 was identified by RNA pull-down and RIP assays. hnRNPA1 is the most ubiquitously expressed member of the HNRNP protein family [36]. hnRNPA1 was involved in diverse aspects of nucleic acid metabolism, such as the packaging of nascent transcripts, alternative splicing, and translational regulation [37]. Dysregulation of hnRNPA1 has been associated with various diseases, including cancer [38]. For example, hnRNPA1 was overexpressed in lung carcinoma, GC, and colorectal cancer [39] and has been correlated with poor prognosis in hepatocellular carcinoma and breast cancer [40, 41]. In this study, GC patients with high levels of hnRNPA1 had unfavorable overall survival in TCGA database, and upregulation of hnRNPA1 protein expression was observed in OIP5-AS1-overexpressing GC cells. Ubiquitination, as one of the major posttranslational protein modifications, has been reported to be involved in various biological processes, including cell survival, differentiation, innate immunity, and adaptive immunity [42, 43]. A growing body of evidence indicates that the hnRNPA1 protein level can be modulated by the ubiquitination pathway [44, 45]. E3 ubiquitin ligase is a critical and heterogeneous enzyme in the ubiquitin–proteasome system [30]. In our study, Trim21 was shown to be downregulated, while hnRNPA1 was found to be upregulated in GC cell lines. Furthermore, Trim21, an E3 ubiquitin ligase, is also bound to HNRNPA1. Recently, studies have found that lncRNAs can interact with ubiquitination-related proteins to regulate the expression of their target proteins [42]. For instance, lncRNA-Fendrr protects against ubiquitination and degradation of the NLRC4 protein through the E3 ubiquitin ligase HERC2 to modulate the pyroptosis of microglia [46]. lncRNA-ASB16-AS1 promotes ubiquitin E3 ligase BTRC-mediated ubiquitination and degradation of LATS1 to inhibit adrenocortical carcinoma cell growth [43]. LncRNA ANCR promoted the metastasis of hepatocellular carcinoma by upregulating hnRNPA1 and inhibiting hnRNPA1 degradation [47]. Consistent with these data, OIP5-AS1 post-translationally promoted hnRNPA1 expression via repressing Trim21-mediated ubiquitination and degradation, thereby facilitating the growth, metastasis, and glycolysis of GC in vivo and in vitro.

Increased glycolysis has recently been identified as a cancer hallmark [48]. PKM is a critical rate-limiting enzyme for the last step of glycolysis [49]. The PKM gene encodes PKM1 and PKM2 by alternative splicing [50]. PKM1 is mainly expressed in high-energy demanding organs, including the brain and muscle tissues, whereas PKM2 is widely expressed in embryonic and tumor tissues [48, 51]. PKM2-induced aerobic glycolysis plays an important role in the occurrence and development of cancer, and the PKM2/PKM1 ratio is upregulated in multiple malignancies [52]. The splicing factorHNRNPA1, which contains two RNA recognition motifs (RRM1 and RRM2), has been linked to cancer progression by facilitating alternative splicing of PKM precursor mRNA exons [49]. This was confirmed in our study to occur in GC. Furthermore, OIP5-AS1 accelerated HNRNPA1-mediated PKM alternative splicing, causing PKM2 formation, which enhanced glucose uptake, lactate production, glycolysis, and glycolytic capacity while reducing ATP production and maximal respiration, thereby promoting growth and metastasis of GC cells.

**Fig. 13:**
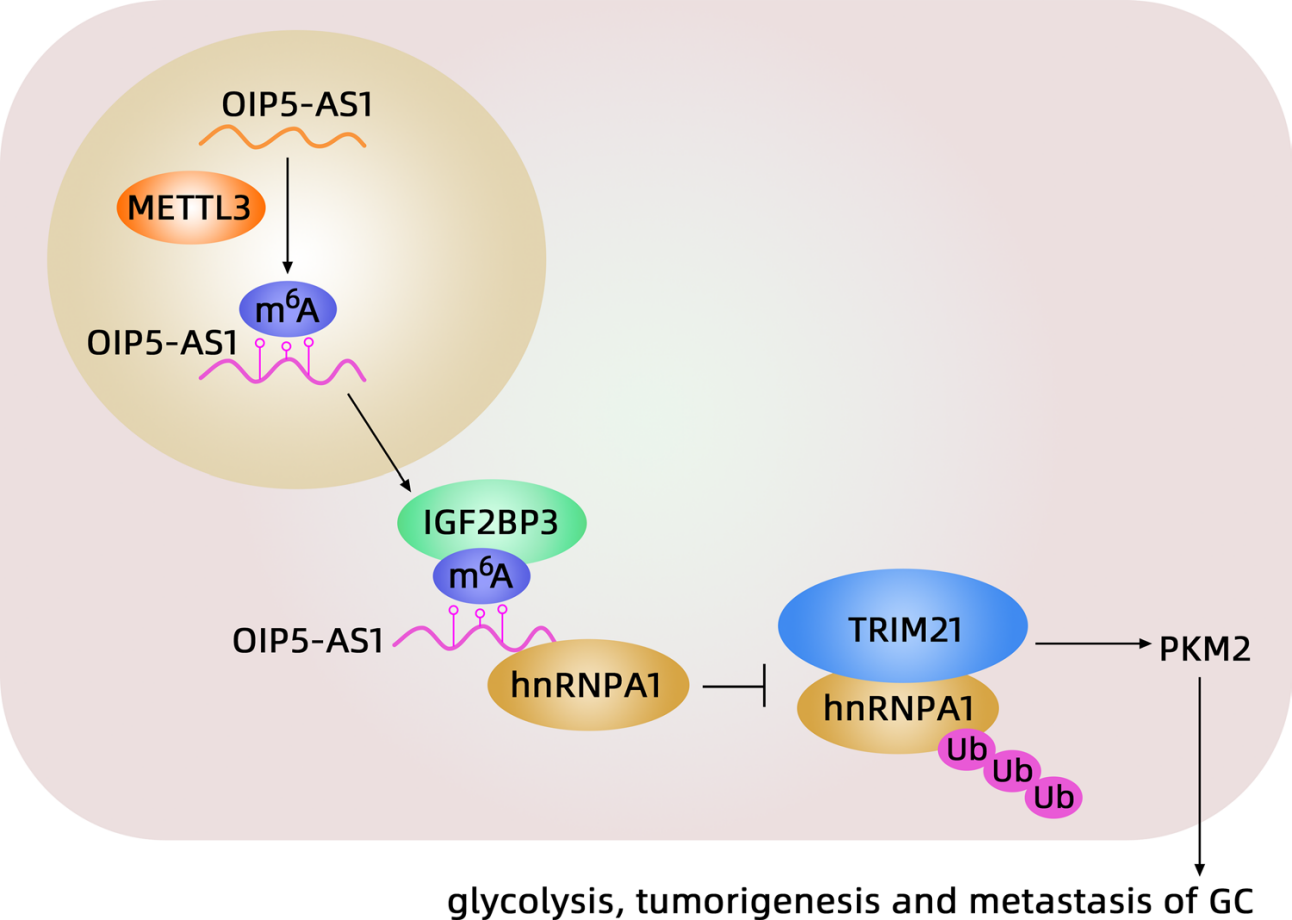
Schematic illustration of the role of OIP5-AS1 in GC

In conclusion, GC patients with high OIP5-AS1 expression have a poor prognosis. OIP5-AS1 stabilized by m6A modification promotes the progression of GC by inhibiting Trim21-mediated ubiquitination and degradation of HNRNPA1, resulting in the formation of PKM2 (Fig. 13). These findings contribute to our understanding of the IGF2BP3/OIP5-AS1/Trim21/hnRNPA1/PKM2 axis in GC and suggest that OIP5-AS1 may open a potential novel perspective for diagnostic and therapeutic regimens in GC.

## Data Availability

All data that support the findings of this study are available from the corresponding authors upon reasonable request.
